# Pertussis Toxin Exploits Host Cell Signaling Pathways Induced by Meningitis-Causing *E. coli* K1-RS218 and Enhances Adherence of Monocytic THP-1 Cells to Human Cerebral Endothelial Cells

**DOI:** 10.3390/toxins8100291

**Published:** 2016-10-13

**Authors:** Laura Julia Starost, Sascha Karassek, Yasuteru Sano, Takashi Kanda, Kwang Sik Kim, Ulrich Dobrindt, Christian Rüter, Marcus Alexander Schmidt

**Affiliations:** 1 Institute of Infectiology, Center of Molecular Biology of Inflammation, Westfälische Wilhelms-Universität Münster, Münster D-48149, Germany; l_star02@uni-muenster.de (L.J.S.); sascha.karassek@hotmail.com (S.K.); rueterc@uni-muenster.de (C.R.); 2 Department of Neurology and Clinical Neuroscience, Yamaguchi University Graduate School of Medicine, Yamaguchi, Ube City 755-8505, Japan; yasuteru@yamaguchi-u.ac.jp (Y.S.); tkanda@yamaguchi-u.ac.jp (T.K.); 3 Pediatric Infectious Diseases Division, Johns Hopkins University School of Medicine, Baltimore, MD 21287, USA; kwangkim@jhmi.edu; 4 Institute of Hygiene, Microbial Genome Plasticity—Molecular Infection Biology, Westfälische Wilhelms-Universität Münster, Münster D-48149, Germany; dobrindt@uni-muenster.de

**Keywords:** pertussis toxin, MAPK p38, neonatal meningitis-causing *E. coli* K1-RS218, NMEC, NF-κB, blood–brain barrier

## Abstract

Pertussis toxin (PTx), the major virulence factor of the whooping cough-causing bacterial pathogen *Bordetella pertussis*, permeabilizes the blood–brain barrier (BBB) in vitro and in vivo. Breaking barriers might promote translocation of meningitis-causing bacteria across the BBB, thereby facilitating infection. PTx activates several host cell signaling pathways exploited by the neonatal meningitis-causing *Escherichia coli* K1-RS218 for invasion and translocation across the BBB. Here, we investigated whether PTx and *E. coli* K1-RS218 exert similar effects on MAPK p38, NF-κB activation and transcription of downstream targets in human cerebral endothelial TY10 cells using qRT-PCR, Western blotting, and ELISA in combination with specific inhibitors. PTx and *E. coli* K1-RS218 activate MAPK p38, but only *E. coli* K1-RS218 activates the NF-κB pathway. mRNA and protein levels of p38 and NF-κB downstream targets including IL-6, IL-8, CxCL-1, CxCL-2 and ICAM-1 were increased. The p38 specific inhibitor SB203590 blocked PTx-enhanced activity, whereas *E. coli* K1-RS218’s effects were inhibited by the NF-κB inhibitor Bay 11-7082. Further, we found that PTx enhances the adherence of human monocytic THP-1 cells to human cerebral endothelial TY10 cells, thereby contributing to enhanced translocation. These modulations of host cell signaling pathways by PTx and meningitis-causing *E. coli* support their contributions to pathogen and monocytic THP-1 cells translocation across the BBB.

## 1. Introduction

Pertussis toxin (PTx), the major virulence factor secreted by the Gram-negative bacterium *Bordetella pertussis*, is systemically distributed and essential for the onset of the acute respiratory disease whooping cough [1,2,3,4,5,6]. Pertussis infections can be asymptomatic or mildly symptomatic, or might present the classic picture of whooping cough over a prolonged period of time. Although a serious disease in infants, pertussis is often unrecognized due to a low awareness and difficulties in diagnosis in early infancy [7]. Recent studies have suggested asymptomatic carriage also in highly vaccinated populations as a major link in the resurgence of pertussis [8,9,10]. Occasionally pertussis can be accompanied by secondary infections and additional serious complications including even neurologic disorders as a concomitant manifestation [11,12,13]. It is debated whether these complications might be associated with PTx, as the toxin enhances the permeability of the blood–brain-barrier (BBB) in vitro and potentially also in vivo. Furthermore, PTx increases the translocation and invasion of immune cells as well as of pathogenic bacteria to the central nervous system (CNS), such as the meningitis-causing bacterium *E. coli* K1 [14,15,16,17]. Some authors even discuss a possible link of subclinical pertussis to the development of multiple sclerosis [18]. Hence, it appears that by facilitating and enhancing the traversal of immune cells and of pathogens across the blood-brain barrier, the activities of PTx during pertussis infection might create a predisposition for additional bacterial infections of the CNS. PTx is a typical A-B_5_ bacterial toxin [19,20] where the enzymatically active A-monomer mediates ADP-ribosylation of the α-subunit of Gi-proteins, while the B-pentamer mediates binding of PTx to target cells, the subsequent toxin uptake [19,20,21,22,23,24], and, furthermore, contributes to the translocation of the A-monomer into the cytosol [21].

*Escherichia coli* K1 strains are major causative agents of meningitis in neonates [25,26]. To evoke acute bacterial meningitis, *E. coli* K1 has to cross the BBB, invade the central nervous system (CNS) and cause inflammation [27,28]. We hypothesized that permeabilization of endothelial barriers by PTx may facilitate translocation not only of immune cells but also of pathogenic bacteria [14,15,16]. In our previous study we demonstrated that PTx induces similar host cell signaling pathways as *E. coli* K1 in endothelial cells of the BBB, thereby enhancing invasion and translocation of *E. coli* K1-RS218 [17]. Paracellular and transcellular transport routes have been suggested as possible pathways for entry of *E. coli* K1 [14,29,30,31,32,33,34,35,36]. In addition, a ‘Trojan horse’ mechanism has been discussed for penetration of CNS-infecting pathogens into the brain [28], where *E. coli* K1 may exploit immune cells as transport vehicles to cross the BBB. Previously we showed, that compared to the laboratory strain C600, *E. coli* K1 was able to survive substantially longer in monocytic cells [15]. Interestingly, PTx enhances the translocation of several types of secondary immune cells across human brain-derived microvascular endothelial cell (HBMEC) barriers [15].

During the extravasation of leukocytes, immune cells egress from blood vessels to invade inflamed tissues. They are activated and recruited in response to pro-inflammatory cytokines and chemokines, whose transcription is regulated mainly by NF-κB, but also by mitogen-activated kinases (MAPK) and, depending on the stimulus or type of signal, especially by the stress kinase p38 MAPK (p38), [37,38,39]. MAPKs can be divided into three major subfamilies: the extracellular signal-regulated kinase (Erk1/2), the c-Jun N-terminal kinase (JNK) and p38 [40,41]. In our previous study [17] we found that PTx and *E. coli* K1-RS218 induce overlapping effects by inhibiting the phosphorylation and thereby the activation of Erk1/2. In this way PTx enhances the dissociation of the adherens junction proteins VE-Cadherin and β-Catenin, which increases the permeability of cell-cell contacts and facilitates paracellular transport [17]. Here, we examined and compared the meningitis-causing *E. coli* K1-RS218 and PTx for their effects on the activation of the p38 and NF-κB pathways, and the transcription of cytokines and chemokines. Furthermore, we examined whether PTx might facilitate binding of immune cells to endothelial cells. We analyzed the effects of PTx on human monocytic THP-1 cells taken as ‘model immune cells’ with respect to endothelial adhesion, elevated production of pro-inflammatory cytokines and activation of STAT3.

## 2. Results

### 2.1. PTx Enhances p38 but Not NF-κB Phosphorylation

**Figure 1:**
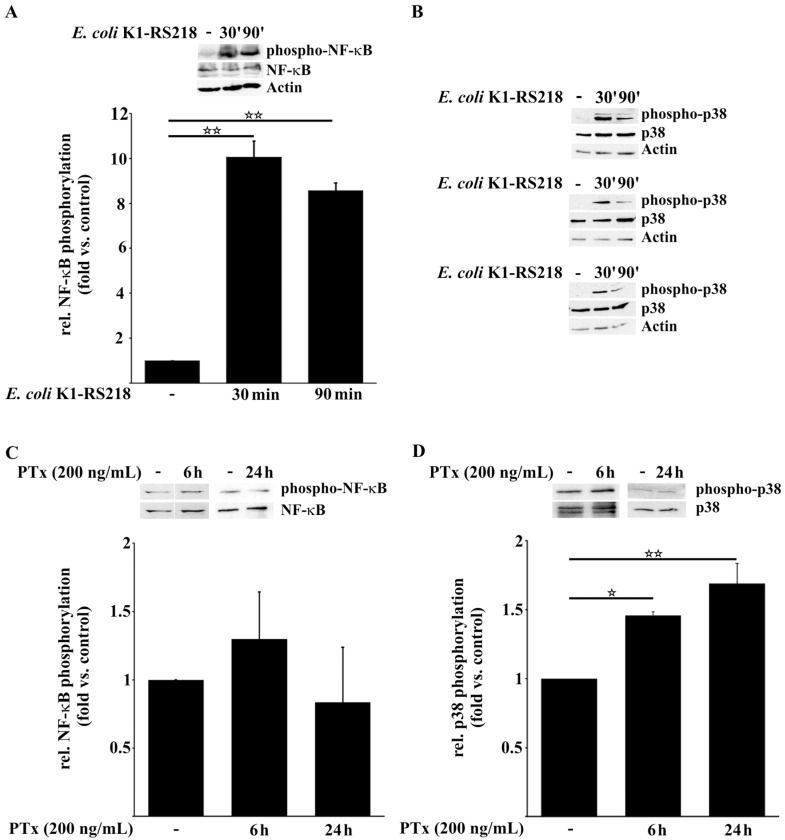
PTx activates p38 MAPK but not NF-κB in human cerebral TY10 endothelial cells. (**A**,**B**) Infection of TY10 cells with *E. coli* K1-RS218 (MOI 100, 30 min and 90 min) increases the phosphorylation of NF-κB at S536 (**A**) and p38 MAPK at T180/Y182 (**B**); (**C**,**D**) Application of PTx (200 ng/mL, 6 h or 24 h) does not affect the phosphorylation of NF-κB (**C**) but significantly increases the phosphorylation of p38 MAPK (**D**). Bars represent the mean ± SEM of at least three independent experiments performed in duplicates. * *p* < 0.05; ** *p* < 0.01 (determined by ANOVA followed by Bonferroni post hoc test). Quantification of relative p38 MAPK activation after *E. coli* K1-RS218 infection was not possible as phosphorylated p38 MAPK signals in untreated cells could not be detected under these conditions. Blot images are representative images of three independent experiments (Figure 1**A**–**D**). (− means without indicated treatment).

Recently we showed that PTx exhibited host cell signaling events similar to those induced by *E. coli* K1-RS218, resulting in increased translocation and invasion of the pathogen across the blood–brain barrier (BBB) [17]. Whereas in our previous study we focused on cell-cell adhesion signaling pathways, here we investigated whether PTx also promotes the activation of the stress-regulated MAPK p38, NF-κB and the transcription of their downstream targets. As primary human cerebral microvascular endothelial cells are not available in sufficient and reliable amounts, we had to resort to a tissue culture model employing stable human brain-derived microvascular endothelial TY10 cells [42,43] as an established surrogate. All assays were done with confluent cells under identical conditions. Western blotting analysis was performed to determine the effects of PTx and *E. coli* K1-RS218 on the activation of the MAPK p38 and NF-κB (Figure 1). Infection of TY10 cells for 30 min or 90 min with the bacteria led to a several fold increase of NF-κB and p38 phosphorylation (Figure 1A,B). Fold values were obtained by comparison of values obtained by Western blotting in stimulated cells vs. values obtained for non-stimulated cells. Values for non-stimulated cells were arbitrarily set to 1. Application of PTx for 6 h and 24 h, however, had no effect on the phosphorylation of NF-κB (Figure 1C). In contrast, we found a significant increase in p38 phosphorylation already 6 h after PTx application (1 vs. 1.46 ± 0.02), which was still detectable after 24 h (1 vs. 1.62 ± 0.14) (Figure 1D). Compared to *E. coli* K1-RS218-induced activation after 30/90 min, p38 phosphorylation induced by PTx was modest but still present after 24 h.

As in our previous study [17] we could not always monitor the effects of PTx and *E. coli* K1-RS218 at the same time (e.g., 2.2) as the bacteria were cytotoxic at incubation times longer than 90 min. In untreated cells, signals of phosphorylated p38 MAPK were not detectable so that, under these conditions, quantification of relative p38 MAPK activation after *E. coli* K1-RS218 infection was not possible.

### 2.2. PTx and E. coli K1-RS218 Enhance mRNA Expression of IL-1β, IL-6, IL-8, (TNFα), CxCL-1, CxCL-2 and ICAM-1

**Figure 2:**
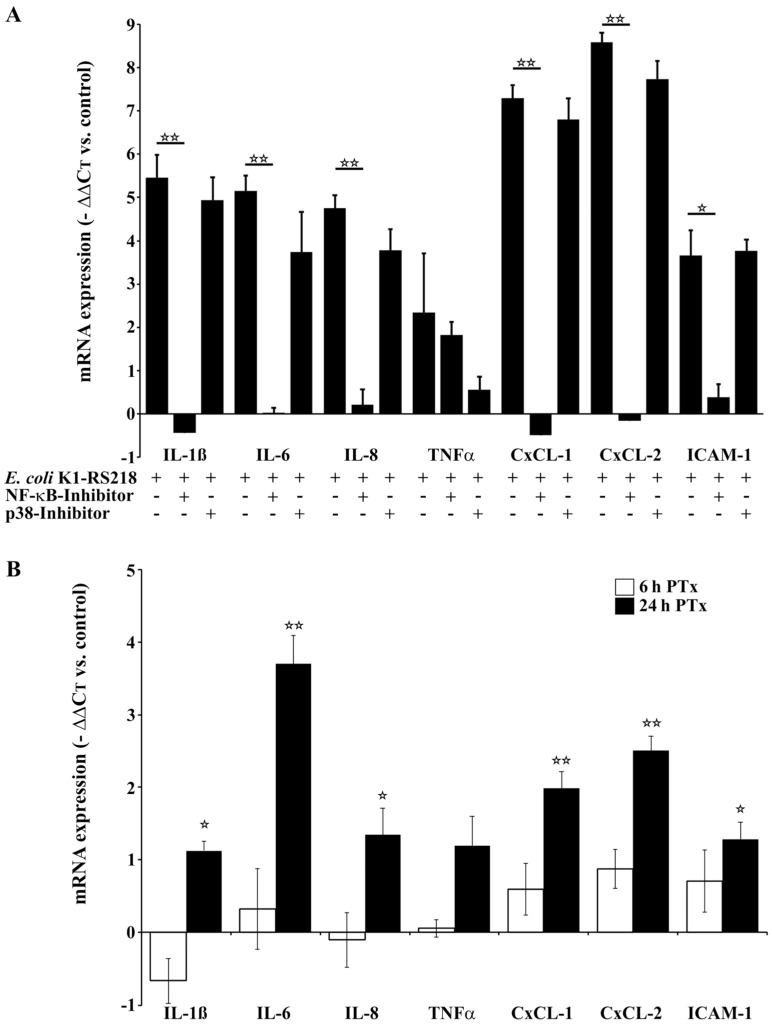
Long-term application of PTx enhances the expression of IL-1β, IL-6, IL-8, CxCL-1, CxCL-2 and ICAM-1 in TY10 cells. (**A**) qRT-PCR analysis of *E. coli* K1-RS218 (MOI 100, 90 min) infected TY10 cells shows significantly increased levels of IL-1β, IL-6, IL-8, CxCL-1, CxCL-2 and ICAM-1 mRNA, but had no effect on TNFα mRNA levels. Application of the NF-κB inhibitor Bay 11-7082 (Bay; 10 µM) completely abolishes increased cytokine and chemokine expression levels, while application of p38 MAPK inhibitor SB203590 (10 µM) had no effect; (**B**) Treatment of TY10 cells with PTx (200 ng/mL, 6 h) does not affect IL-1β, IL-6, IL-8, TNFα, CxCL-1, CxCL-2 and ICAM-1 mRNA levels. In contrast, 24 h application of PTx (200 ng/mL) significantly enhances the mRNA levels of all examined genes. Bars represent the mean ± SEM of at least three independent experiments performed in duplicates. * *p* < 0.05; ** *p* < 0.01 (determined by ANOVA followed by Bonferroni post hoc test). (**A**) Only significant differences are shown comparing *E. coli* K1-RS218 samples with *E. coli* K1-RS218 + Bay 11-7082/SB203590 inhibitors; (**B**) Only the significances treated vs. untreated cells are given. +: with indicated treatment; −: without indicated treatment.

Next, we used qRT-PCR to analyze the influence of PTx and *E. coli* K1-RS218 on downstream targets of p38 and NF-κB in human TY10 cerebral endothelial cells, as these might play a role in inflammation, immune cell attraction and adhesion. For this, we measured transcription levels of the cytokines IL-1β, IL-6, IL-8 and TNFα, of the chemokines CxCL-1 and CxCL-2, and of the adhesion molecule ICAM-1 (Figure 2). *E. coli* K1-RS218 infections led to significantly elevated mRNA levels of all genes examined except TNFα, which was only slightly enhanced. However, this might be a specific feature of TY10 cells (Figure 2A). To further elucidate the contribution of NF-κB and p38, we used either the specific NF-κB inhibitor Bay 11-7082 or the specific p38 inhibitor SB203580 (Figure 2A). These inhibitors did not show any cytotoxic activities under the conditions used in our experiments (24 h incubation with the indicated concentrations) (data not shown). Also, proliferation of cells was not affected. Inhibition of p38 had no significant effect on the transcription levels of the chemo- and cytokines analyzed in this study with the exception of TNFα expression, which was also affected by the p38 inhibitor SB203580 (10 µM) (Figure 2A). In contrast, inhibition of NF-κB using the Bay 11-7082 inhibitor (10 µM) completely abolished *E. coli* K1-RS218-induced transcription, which confirmed that *E. coli* K1-RS218 triggers the NF-κB signaling pathway [17].

Application of PTx showed no significant alterations of transcription rates at 6 h (Figure 2B); however, incubation for 24 h led to a significant increase of transcription (Figure 2B). The elevated mRNA levels were clearly dependent on the catalytic A-monomer (S1 of PTx) as the B-oligomer alone did not at all influence transcription levels (Figure S1). Interestingly, *E. coli* K1-RS218 also enhanced cytokine and chemokine transcription much more quickly than PTx, for which a significant enhancement of cytokine and chemokine transcription was only found at 24 h of incubation.

### 2.3. PTx Increases IL-6, IL-8, CxCL-1, CxCL-2 and ICAM-1 Protein Levels

**Figure 3:**
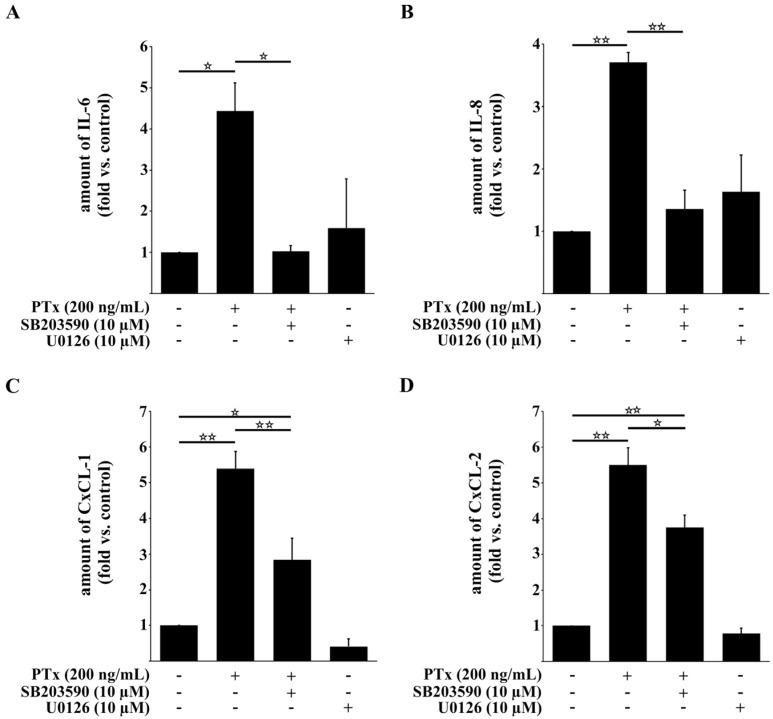
PTx increases the secretion of IL-6, IL-8, CxCL-1, and CxCL-2 in TY10 cells. ELISA analysis of TY10 supernatants shows that application of PTx (200 ng/mL, 24 h) significantly increases the amount of IL-6, IL-8, CxCL-1 and CxCL-2. Parallel application of p38 MAPK inhibitor SB203590 (10 µM, 24 h) completely inhibits secreted protein levels of IL-6 and IL-8, but has only a partial inhibitory effect on secreted protein levels of CxCL-1 and CxCL-2. Bars represent the mean ± SEM of at least three independent experiments performed in duplicates. * *p* < 0.05; ** *p* < 0.01 (determined by ANOVA followed by Bonferroni post hoc test). **A**: amount of IL-6; **B**: amount of IL-8; **C**: amount of CxCL-1; **D**: amount of CxCL-2; +: with indicated treatment; −: without indicated treatment.

To further confirm that prolonged PTx application is able to drive the expression of certain cytokines, chemokines and cell adhesion receptors, we analyzed supernatants of TY10 cells by ELISA (Figure 3) and Western blotting (data not shown). No IL-1β and TNFα was detectable in the supernatants of TY10 cells following the application of PTx for 24 h. However, PTx incubation led to elevated levels of all other chemo- and cytokines tested in this study, thus confirming our qRT-PCR results (Figure 3A–D). Expression of ICAM-1 on TY10 cells was enhanced by PTx treatment (24 h) (Figure S2). Inhibition of p38 by the specific inhibitor SB203580 showed that upregulation of gene transcription and protein secretion by PTx involves the activation of p38. Furthermore, as it had been reported recently that PTx inhibits the Erk1/2 pathway [17] we included the Erk1/2 inhibitor U0126, which, in case of involvement of the Erk1/2 pathway, should have a similar effect as PTx. However, U0126 had no effect on the secretion of IL-6, IL-8, CxCL-1 and CxCL-2 thus ruling out an involvement of Erk1/2 signaling. These inhibitor studies showed that inhibition of p38 abolished the induction of increased levels of IL-6 and IL-8 by PTx. Furthermore, the elevation of CxCL-1 and CxCL-2 levels was significantly reduced compared to PTx treatment, but levels were still higher compared to the untreated control group, possibly pointing to the involvement of a so-far unknown pathway influenced by PTx.

Our results show that PTx and *E. coli* K1-RS218 clearly differ with respect to the activation of the p38 pathway to upregulate protein expression of pro-inflammatory cytokines and chemokines.

### 2.4. PTx Enhances the Adherence of Monocytic THP-1 Cells to Cerebral Endothelial TY10 Cells Involving the Activation of the p38 Pathway

**Figure 4:**
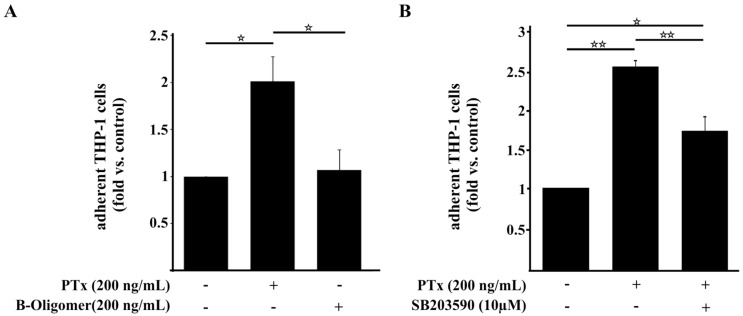
PTx enhances the adherence of THP-1 cells to TY10 cells in part via p38. (**A**) Treatment of TY10 cells with PTx (200 ng/mL, 24 h) significantly enhances the number of bound THP-1 cells to TY10 cells. In contrast, application of the B-Oligomer (200 ng/mL, 24 h) had no significant effect on THP-1 adherence; (**B**) Application of p38 MAPK inhibitor SB203590 (10 µM, 24 h) significantly decreases the induced adherence of THP-1 to TY10 cells by PTx. Bars represent the mean ± SEM of at least three independent experiments performed in duplicates. * *p* < 0.05; ** *p* < 0.01 (determined by ANOVA followed by Bonferroni post hoc test). +: with indicated treatment; −: without indicated treatment.

As pro-inflammatory cytokines such as IL-6 and IL-8, chemokines such as CxCL-1 and CxCL-2 and adhesion molecules like ICAM-1 are involved in the activation and recruitment of immune cells to endothelial cells [37,39,44,45,46], we next investigated whether PTx affects the adherence of human immune cells to cerebral endothelial TY10 cells. For this we used human monocytic THP-1 cells in a tissue culture model system (Figure 4). As illustrated in Figure 4A we detected a significant increase in adherence of THP-1 cells to endothelial TY10 monolayers, which had been treated previously for 24 h with PTx (1 vs. 2.4 ± 0.37). In accordance with the qRT-PCR results, incubation with the PTx B-oligomer had no effect whatsoever on the adherence of THP-1 cells (Figure 4A). Next, we analyzed the effect of the p38 MAPK inhibitor SB203580 on the binding of THP-1 to TY10 cells. Inhibition of p38 MAPK significantly decreased the number of bound THP-1 cells compared to the PTx-treated samples without p38 inhibitor (2.5 ± 0.07 vs. 1.7 ± 0.17). This result is in agreement with our ELISA data, which showed an involvement of p38 activity in the secretion of cytokines and chemokines (Figure 3). The residual increase of THP-1 cell binding to stimulated TY10 monolayers after p38 inhibition might be attributed to increased levels of the chemokines CxCL-1 and CxCL-2, which inhibition were slightly elevated even after p38 (Figure 3C,D).

In addition, PTx also enhanced the expression of the intercellular adhesion molecule 1 (ICAM-1, CD54) on TY10 cells (Figure S2).

### 2.5. PTx-Treated TY10 Cells Induce the Expression of Pro-Inflammatory Cytokines and the Activation of STAT3 in THP-1 Cells

**Figure 5:**
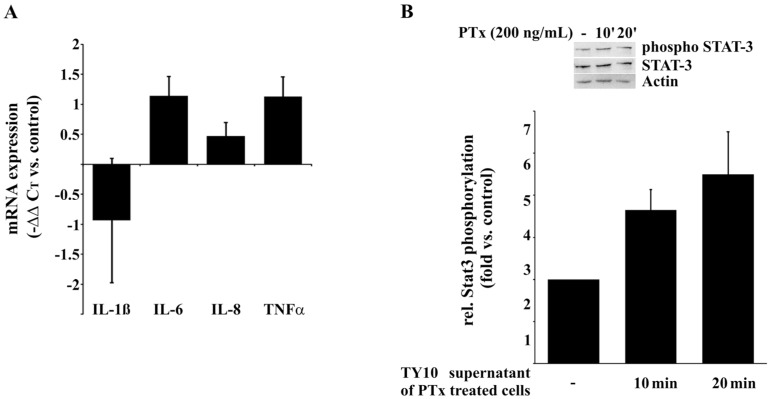
The supernatant of PTx-treated TY10 cells induces enhanced expression of cytokines and phosphorylation of STAT3 in THP-1 cells. (**A**) Differentiated THP-1 cells (PMA, 1 µg/mL, 24 h) showed increased mRNA expression of pro-inflammatory cytokines IL-6 and TNFα after 3 h of incubation in the supernatant collected from TY10 cells treated with PTx (200 ng/mL, 24 h); (**B**) Incubation of differentiated THP-1 cells with supernatant of PTx-treated TY10 cells (200 ng/mL, 24 h) for 10 and 20 min results in increased phosphorylation of STAT3. Bars represent the mean ± SEM of at least three independent experiments performed in duplicates. However, using ANOVA followed by Bonferroni post hoc test statistic significances could not be determined. (Figure 5B: −: no treatment).

Next, we investigated whether PTx not only enhances the adherence of THP-1 cells to endothelial TY10 cells but, due to the enhanced secretion of CxCL-1 and CxCL-2, might also induce the secretion of pro-inflammatory cytokines in differentiated THP-1 cells. For this we examined the effect of the supernatant of PTx-treated TY10 cells on differentiated THP-1 cells for the activation of pro-inflammatory cytokines. In addition, we monitored the phosphorylation of STAT3, which is known as an ‘anti-inflammatory response’ in macrophages [47]. The mRNA levels of IL-6 and TNFα were significantly enhanced following incubation of THP-1 cells for 3 h with the supernatant of PTx-treated cells, while IL-1β and IL-8 levels remained unaffected (Figure 5A). In addition, phosphorylation of STAT3 was significantly enhanced after incubation with the supernatant of PTx-treated TY10 cells, indicating that the THP-1 cells were activated (Figure 5B).

## 3. Discussion

Pertussis toxin (PTx) is the major virulence factor of *Bordetella pertussis*, the causative agent of the acute respiratory disease whooping cough. Although vaccination has been quite successful in the past, the recent resurgence of pertussis cases also in vaccinated populations is of great concern. It appears that the acellular vaccine is able to prevent disease but not infections. PTx is a systemically distributed toxin and has been held responsible for multiple manifestations throughout the body. Many studies have convincingly shown that PTx-induced permeabilization of endothelial barriers promotes disease in various models e.g. experimental autoimmune encephalomyelitis (EAE). As diagnosis of asymptomatic or mild pertussis is still problematic, particularly in neonates, we hypothesized that in the case of unrecognized pertussis infections PTx might promote the traversal of hematogenic pathogens such as meningitis-causing *E. coli* K1 strains across the BBB [14,15,16]. We have recently shown by employing an established in vitro model that interactions of the *Bordetella pertussis* exotoxin pertussis toxin (PTx) with human cerebral endothelial cells (TY10) [42,43] activate important signaling pathways that are also induced by *E. coli* K1-RS218 during invasion and translocation of the pathogen across the BBB [17]. By enforcing these signaling events PTx increases the rate of translocation and invasion of bacterial pathogens and immune cells [15,16]. Here, we extended our investigation and compared the effects of PTx and *E. coli* K1-RS218 on the activation of the NF-κB and p38 signaling pathways and investigated the expression of cytokines and chemokines, which are crucial for immune cell attraction, activation and adhesion. Our data show that both PTx and *E. coli* K1-RS218 induce the activation of p38 MAPK (Figure 1B,D), but that activation of NF-κB occurs only in response to *E. coli* K1-RS218 (Figure 1A,C). While infections with *E. coli* K1-RS218 led to a fast and robust response application of PTx showed a modest but prolonged increase in p38 MAPK activity up to 24 h. Unfortunately, due to substantial cytotoxicity, incubating endothelial cells with *E. coli* K1-RS218 for longer than 90 min was not possible. The modest increase in p38 activity might be responsible for the enhanced, but delayed transcription of IL-1β, IL-6, IL-8, TNFα, CxCL-1, CxCL-2 and ICAM-1 genes only after 24 h of incubation with PTx (Figure 2). Interestingly, the *E. coli* K1-RS218-induced gene transcription of IL-1β, IL-6, IL-8, TNFα, CxCL-1, CxCL-2 and ICAM-1 depended completely on NF-κB activity (Figure 2A) while PTx-induced gene transcription and secretion depended on p38 and potentially also other non-identified pathways. Whether the activation of the p38 pathway provides a potential advantage for these bacterial pathogens in bacterial meningitis is not fully understood. It has been reported that for bacterial meningitis the triad of bacterial invasion, NF-κB activation, and leucocyte transmigration is important where p38 activation might also play a role [48,49]. However, since CxCL-1 and CxCL-2 protein levels were only partially dependent on p38 activity, there might be another yet unrecognized pathway affected by PTx. The signal transducer and activator of transcription (STAT) pathway might be a potential candidate, since STAT proteins are known to regulate the activation of CxCL-1 and CxCL-2 [44]. In addition, it was recently shown that PTx induces the activation of STAT3 in cerebral endothelial TY10 cells [17].

As IL-6, IL-8, CxCL-1, CxCL-2 and ICAM-1 are involved in the activation and recruitment of immune cells [37,38,44,45,46], we analyzed the effect of PTx on the adherence of monocytic THP-1 cells to TY10 cells. Incubation with PTx elevated the secretion of pro-inflammatory cytokines and chemokines and enhanced the adherence of THP-1 cells to TY10 monolayers. This is further promoted by the increased expression of ICAM-1. In agreement with our qRT-PCR data, this effect is due to activities of PTx-S1 and independent of the PTx B-oligomer. Moreover, p38 inhibition led to a significant decrease in adherent THP-1 cells corresponding to our ELISA data. Since CxCL-1 and CxCL-2 protein levels were only partially dependent on p38 activity, this might be a reason for the residual increase in bound THP-1 cells after inhibition of p38.

Furthermore, the effect of the supernatant of PTx-treated TY10 cells on THP-1 cells was examined. The enhanced expression of IL-6 and TNFα in THP-1 cells might lead to a positive feedback loop. As shown previously, PTx enhances the expression of pro-inflammatory cytokines in TY10 cells [17], which activated immune cells. These immune cells increased their own cytokine expression due to the cytokines in the supernatant of TY10 cells, which could in turn activate additional immune cells. In addition, phosphorylation of STAT3 in differentiated THP-1 cells seems to be slightly enhanced after incubation with the supernatant of PTx-treated TY10 cells. Several studies of other groups indicated that STAT3 is activated in response to elevated cytokine levels and mediates an ‘anti-inflammatory response’, which should typically prevent enhanced cytokine levels [47,50,51,52]. This might be due to an early and a late response to enhanced cytokine levels in the supernatant of PTx-treated TY10 cells. The early response of THP-1 cells already after some minutes aims to have an anti-inflammatory effect, while the late response leads to the recruitment of further immune cells.

It appears that the PTx-induced synergistic effects on gene transcription investigated here are solely dependent on the activity of the A-protomer and not influenced by B-oligomer activities as has also been found for e.g. the development of encephalitogenic T cells [53]. In future studies it might also be interesting to investigate the putative effects of PTx on the c-Jun-*N*-terminal kinase (JNK) pathway.

In summary, we provide evidence that PTx induces the p38 MAPK pathway, which leads to elevated expression of cytokines and chemokines. Secretion of these proteins contributes to the adherence of THP-1 cells to human cerebral endothelial TY10 cell monolayers which is further enhanced by the PTx-induced elevated expression of ICAM-1. In addition this appears to induce further cytokine production in THP-1 cells resulting in a positive feedback loop. Previous studies [15] had demonstrated that *E. coli* K1 enters immune cells such as monocytes and macrophages to a significantly larger extent than the non-pathogenic *E. coli* strain C600 and remains viable for at least 48 h. Furthermore, it had been shown that the translocation of *E. coli* K1 inside HL60-derived macrophages or U937 monocytes was significantly enhanced upon parallel treatment with PTx [15]. In the present study we demonstrated that the adherence of human monocytic THP-1 cells to human cerebral endothelial TY10 cells (as a prerequisite for translocation) is significantly enhanced due to the activities of PTx. Altogether, based on our findings, we propose that PTx enhances the translocation of *E. coli* K1-RS218 possibly including also the Trojan horse mechanism from immune cell extravasation after long-term exposure to the toxin. In contrast, short-term exposure of PTx does not affect gene regulation significantly, but enhances invasion and translocation of *E. coli* K1-RS218 via dissociation of VE-cadherin and β-catenin at adherens junctions [17].

Additional studies are needed on the molecular mechanism by which PTx increases the expression of these genes and the adherence of THP-1 to cerebral endothelial TY10 cells. This would contribute to the development of novel strategies for interfering with potential secondary infections arising from the PTx-induced permeabilization of the BBB for pathogens and immune cells.

## 4. Materials and Methods

### 4.1. Chemicals, Antibodies and Bacterial Strains

All chemicals were acquired from Sigma unless stated otherwise. Antibodies were obtained from Cell Signaling (Frankfurt, Germany) with the exception of α-ICAM-1 (R&D Systems, Minneapolis, MN, USA) and α-Actin (Sigma, Taufkirchen, Germany). Specific inhibitors were purchased from InvivoGen (Toulouse, France; MAPK p38: SB203590) [54], Merck (Darmstadt, Germany; NF-κB: Bay 11-7082) [55] or Cell Signaling (Frankfurt, Germany; Erk1/2: U0126) [56]. Pertussis toxin and the PTx B-oligomer were purchased from Calbiochem (Merck, Darmstadt, Germany). *E. coli* K1-RS218 (O18ac:K1:H7) is the prototype neonatal meningitis *E. coli* strain (NMEC) and has been obtained from a clinical isolate from the cerebrospinal fluid of a newborn with meningitis [57].

### 4.2. Cell Culture

Human brain-derived microvascular endothelial TY10 cells [42,43] were cultured in Endopan 3 medium (Pan-Biotech, Aidenbach, Germany) supplemented with 20% FBS (Sigma, Taufkirchen, Germany) at 33 °C for proliferation and 37 °C for maturation. The TY10 human brain microvascular endothelial cells have been generated by using transfection with a temperature-sensitive SV40 large T-antigen (tsA58) leading to an immortalization of the cells at 33 °C which is used for proliferation. Upon shifting the cells to 37 °C the tsA58 protein is inactivated and cell growth is arrested. After further cultivation of the conditional immortal cells for 96 h (maturation/differentiation), the cells resemble the parental non-immortalized cells retaining their in vivo BBB functions. The cell line was subcultured up to 80% confluence before passaging. THP-1 cells [58] were maintained in RPMI medium (Sigma, Taufkirchen, Germany) with 10% FBS (Sigma, Taufkirchen, Germany) at 37 °C.

### 4.3. Western Blotting

TY10 cells were grown at 33 °C in 60 mm dishes up to confluence and then matured at 37 °C for 96 h to adopt their parental phenotype [42,43]. Following stimulation with PTx (200 ng/mL, 6/24 h) or infection with *E. coli* K1-RS218 (MOI 100, 90 min) cells were washed with ice cold phosphate-buffered saline (PBS) and lysed in RIPA buffer (50 mM Tris/HCl, pH 7.4, 150 mM NaCl, 0.1% SDS, 0.5% sodium deoxycholate, phosphatase and protease inhibitors). The proteins (20 µg total protein) were separated by SDS-PAGE and analyzed by Western blotting. For this, we used a tank-blot system (11.5 mM Tris, pH 7.8) and transferred the proteins for 2 h at 200 mA. Subsequently, the nitrocellulose membranes were incubated for 1 h in a suspension of TBS-T buffer (150 mM NaCl, 20 mM Tris, 0.05% Tween-20, pH 7.4) containing 5% low-fat milk powder to block free binding sites of the nitrocellulose. Then the membranes were washed in TBS-T and incubated with the first antibody in TBS-T overnight. Depending on the antigen to be detected specific rabbit monoclonal antibodies were applied at a dilution of 1:1000 (Cell Signaling Technology, Frankfurt, Germany). Subsequently, the membranes were washed three times for 5 min with TBS-T and incubated at room temperature for 1 h with the horseradish peroxidase-conjugated secondary antibody dilution in TBS-T employing goat-anti-rabbit IgG antibodies at a dilution of 1:10,000 (Dianova, Hamburg, Germany). After washing three times for 5 min in TBS-T the membranes were developed using the Pierce ECL Western Blotting Substrate (Thermo Scientific, Rockford, IL, USA). The Western blots were analyzed using a Lumi-Imager F1™ device (Roche, Mannheim, Germany) and evaluated by densitometry with the Lumi-Analyst™ Software version 3.1 (Roche, Mannheim, Germany).

### 4.4. qRT-PCR

Confluent TY10 cells grown in 60 mm dishes were treated with PTx (200 ng/mL, 6 h/24 h), *E. coli* K1-RS218 (MOI 100, 90 min) and 10 µM NF-kB inhibitor (Bay 11-7082, Merck, Darmstadt, Germany) or 10 µM SB203590 p38 MAP kinase inhibitor (InvivoGen, Toulouse, France). Total RNA was directly isolated using RNeasy Mini Kit (Qiagen, Hilden, Germany) according to the manufacturer’s instructions. RNA concentration was measured via NanoDrop and purity was controlled by A260/280. RNA was stored at −80 °C until further use. 1 µg RNA was reverse transcribed with Primescript RT Reagent Kit (TaKaRa, St.-Germain-en-Laye, France) and Oligo-dT primers. Quantitative RT-PCR was performed using the SYBR Premix Ex Taq II Kit (TaKaRa, St.-Germain-en-Laye, France). Real-Time PCR conditions were as follows: 95 °C for 5 min, 40 cycles: 95 °C for 10 s, 60 °C for 30 s. The fold increase in expression of mRNA was normalized to the expression of hypoxanthine phosphoribosyltransferase (HPRT). The quantitative real-time data shown are the results of three independent experiments, each performed in duplicate using the primers listed in Table 1. Intron spanning primer for each gene were chosen from the Roche universal probe library and tested for specificity and efficiency vs. the reference gene before use in the experiments.

### 4.5. Enzyme-Linked Immunosorbent Assay (ELISA)

TY10 cells were grown in 24-well plates up to confluence and differentiated for 96 h as described in 4.2 and 4.3. Following 24 h stimulation with PTx (200 ng/mL), Bay 11-8092 (10 µM) or SB203590 (10 µM), ELISA for IL-1β, IL-6, IL-8, TNFα (eBioscience Affymetrix ‘Ready-Set-Go’; Frankfurt, Germany), CxCL-1 (R&D Systems; Minneapolis, MN, USA) and CxCL-2 (Peprotech, Hamburg, Germany) were performed according to the manufacturer’s instructions.

### 4.6. Adherence Assay

For adhesion assays coverslips were coated with collagen using rat tail collagen I (Thermo Fisher Scientific, Darmstadt, Germany) according to the supplier’s recommendations with some modifications. Briefly, the 3 mg/mL collagen I solution was diluted in sterile PBS to 50 µg/mL. Coverslips were coated at 5 µg/cm^2^ and incubated for 30 min at 37 °C. Then, the coverslips were washed three times with sterile PBS followed by seeding of the cells. Confluent TY10 cells grown on collagen-coated coverslips were stimulated with PTx (200 ng/mL), PTx-B (200 ng/mL), or SB203590 (10 µM) for 24 h. Meanwhile 1 × 10^6^ THP-1 cells were washed with PBS and incubated in 1 mM Celltracker™ fluorescent probes (Life Technologies, Darmstadt, Germany) diluted in PBS for 30 min at 37 °C. After three washing steps with PBS, the THP-1 cells were resuspended in 4 ml FBS-free cell culture medium and incubated for 24 h at 37 °C. Afterwards, the cells were resuspended in 1 mL TY10 basal medium and 50 µL cell suspension were added to the stimulated TY10 cells followed by incubation for 1 h. After three extensive washing steps with PBS the samples were mounted with Fluorescent Mounting medium (DakoCytomation, Hamburg, Germany). The slides were imaged with the BZ-II Viewer software of the Biorevo BZ-9000 microscope (Keyence, Neu-Isenburg, Germany) and evaluated with the BZ-II Analyzer (Keyence, Neu-Isenburg, Germany).

### 4.7. Activation of THP-1 Cells

TY10 cells were grown at 33 °C in 60 mm dishes up to confluence, differentiated for 96 h as described above and stimulated with PTx (200 ng/mL, 24 h). Meanwhile, 1 × 10^6^ THP-1 cells were differentiated with PMA (1 µg/mL, 24 h; Sigma, Taufkirchen, Germany). Subsequently these THP-1 cells were washed and incubated with the supernatant of the stimulated TY10 cells (10/20 min). Lysed cells were then subjected to SDS-PAGE and Western Blotting.

### 4.8. Statistical Analysis

Statistical significances were determined using ANOVA followed by Bonferroni post hoc tests, except for data presented in Figures S2 where ANOVA was not applicable and a two-tailed Student’s *t*-test was used.

## Figures and Tables

**Table 1:** Primer pairs used in this study for qRT-PCR.

Primer	F: Forward R: Reverse	Sequence (5′ -> 3′)
HPRT	F	GCCAGACTTTGTTGGATTTG
HPRT	R	CTCATCTTAGGCTTTGTATTTTG
IL-1β	F	TACCTGTCCTGCGTGTTGAA
IL-1β	R	TCTTTGGGTAATTTTTGGGATCT
IL-6	F	GTCCTTCGGGCTCCTTGT
IL-6	R	CAGCACAGCAGAGACAGGAC
IL-8	F	GAGCACTCCATAAGGCACAAA
IL-8	R	ATGGTTCCTTCCGGTGGT
TNFα	F	GTCCAGGCTTGTCCTGCTAC
TNFα	R	AGTCCTGAGGCCTGTGTTTG
CxCL-1	F	GCTGAACAGTGACAAATCCAAC
CxCL-1	R	CTTCAGGAACAGCCACCAGT
CxCL-2	F	CCCATGGTTAAGAAAATCATCG
CxCL-2	R	CTTCAGGAACAGCCACCAAT
ICAM-1	F	CCTTCCTCACCGTGTACTGG
ICAM-1	R	AGCGTAGGGTAAGGTTCTTGC

## Supplementary Materials

The following are available online at www.mdpi.com/2072-6651/8/10/291/s1, Figure S1: Long-term application of the PTx B-Oligomer has no effect on the expression of IL-1β, IL-6, IL-8, CxCL-1, CxCL-2 and ICAM-1 in TY10 cells. qRT-PCR analysis of TY10 cell treated with B-oligomer (200 ng/mL, 24 h) does not affect IL-1β, IL-6, IL-8, TNFα, CxCL-1, CxCL-2 and ICAM-1 mRNA levels. Bars represent the mean ± SEM of at least three independent experiments performed in duplicates. Statistical significance was determined by ANOVA followed by Bonferroni post hoc test, Figure S2: PTx increases ICAM-1 expression in cerebral TY10 endothelial cells. Western blotting analysis of whole TY10 cell lysates after PTx application (200 ng/mL, 24 h) show increased ICAM-1 protein expression levels. Bars represent the mean ± SEM of three independent experiments performed in duplicates. * *p* < 0.05 (determined by the two-tailed Student’s *t*-test). +: with PTx treatment; −: without PTx treatment.

## Abbreviations

The following abbreviations are used in this manuscript:

BBB	Blood-brain barrier
CNS	central nervous system
ELISA	enzyme-linked immunosorbent assay
Erk1/2	extracellular signal-regulated kinase
FBS	fetal bovine serum
HBMEC	human brain-derived microvascular endothelial cell
HPRT	hypoxanthine phosphoribosyltransferase
ICAM-1	intercellular adhesion molecule 1
IL	interleukin
JNK	c-jun *N*-terminal kinase
PBS	phosphate-buffered saline
PMA	phorbol 12-myristate 13-acetate
PTx	pertussis toxin
MAPK	mitogen-activated protein kinase
NF-κB	nuclear factor kappa-light-chain-enhancer of activated B cells
NMEC	neonatal menigitis-causing *E. coli*
qRT-PCR	quantitative real time polymerase chain reaction
RIPA	radioimmunoprecipitation
SDS-PAGE	sodium dodecyl sulfate polyacrylamide gel electrophoresis
STAT3	signal transducer and activator of transcription 3
TNFα	tumor necrosis factor α
