# Supplementary material for: Pertussis Toxin Exploits Host Cell Signaling Pathways Induced by Meningitis-Causing E. coli K1-RS218 and Enhances Adherence of Monocytic THP-1 Cells to Human Cerebral Endothelial Cells
Source: Toxins (Basel). 2016 Oct 13;8(10):291. doi: 10.3390/toxins8100291 (PMC5086651; doi:10.3390/toxins8100291)
Supplement: Supplementary file 1 [file toxins-08-00291-s001.pdf]

Supplementary Materials: Pertussis Toxin Exploits Host Cell Signaling Pathways Induced by Meningitis-Causing *E. coli* K1-RS218 and Enhances Adherence of Monocytic THP-1 Cells to Human Cerebral Endothelial Cells

Laura Julia Starost, Sascha Karassek, Yasuteru Sano, Takashi Kanda, Kwang Sik Kim, Ulrich Dobrindt, Christian Rüter and Marcus Alexander Schmidt

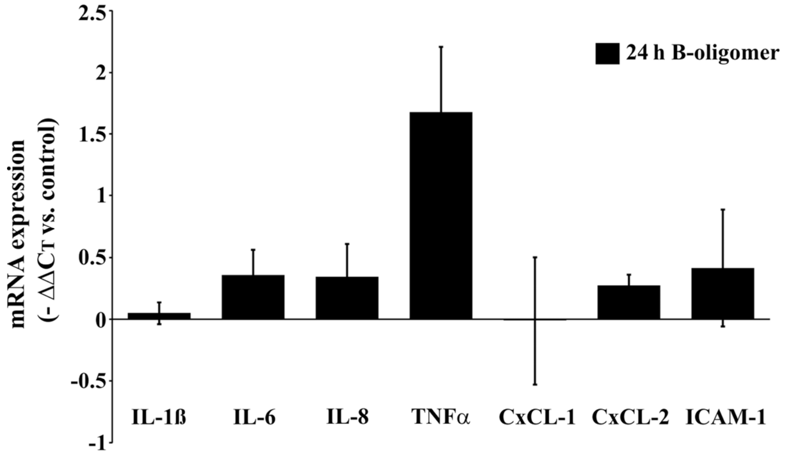

Figure S1. Long-term application of the PTx B-Oligomer has no effect on the expression of IL-1β, IL-6, IL-8, CxCL-1, CxCL-2 and ICAM-1 in TY10 cells. qRT-PCR analysis of TY10 cell treated with B-oligomer (200 ng/mL, 24 h) does not affect IL-1β, IL-6, IL-8, TNFα, CxCL-1, CxCL-2 and ICAM-1 mRNA levels. Bars represent the mean ± SEM of at least three independent experiments performed in duplicates. Statistical significance was determined by ANOVA followed by Bonferroni post hoc test.

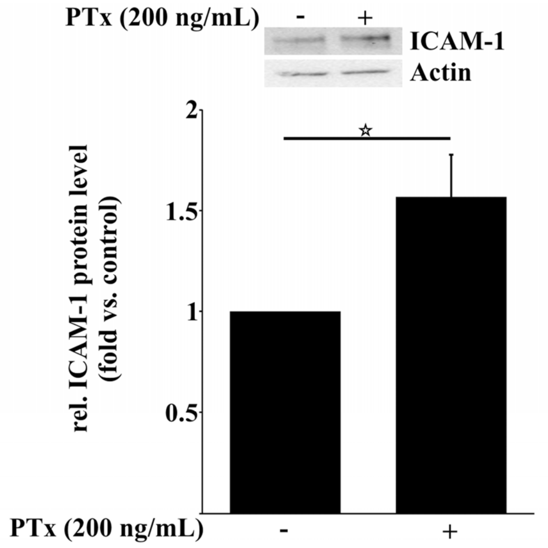

Figure S2. PTx increases ICAM-1 expression in cerebral TY10 endothelial cells. Western blotting analysis of whole TY10 cell lysates after PTx application (200 ng/mL, 24 h) show increased ICAM-1 protein expression levels. Bars represent the mean ± SEM of three independent experiments performed in duplicates. * $p < 0.05$ (determined by the two-tailed Student's *t*-test). +: with PTx treatment; −: without PTx treatment.
